# Healthcare utilization and out-of-pocket expenditures associated with depression in adults: a cross-sectional analysis in Nepal

**DOI:** 10.1186/s12913-020-05094-9

**Published:** 2020-03-25

**Authors:** Selina Rajan, Sujit D. Rathod, Nagendra P. Luitel, Adrianna Murphy, Tessa Roberts, Mark J. D. Jordans

**Affiliations:** 1 Department of Health Services Research and Policy, London School of Hygiene & Tropical Medicine, London, UK; 2 Department of Population Health, London School of Hygiene & Tropical Medicine, London, UK; 3 Research Department, Transcultural Psychosocial Organization Nepal, Kathmandu, Nepal; 4 Centre for Global Chronic Conditions, London School of Hygiene & Tropical Medicine, London, UK; 5 Health Service & Population Research Department, Institute of Psychiatry, Psychology and Neuroscience, King’s College London, 16 De Crespigny Park, Camberwell, London, SE5 8AF UK

**Keywords:** Major depressive disorder, Nepal, Health services, Hospitalization, Out-of-pocket expenditures

## Abstract

**Background:**

Despite attempts to improve universal health coverage (UHC) in low income countries like Nepal, most healthcare utilization is still financed by out-of-pocket (OOP) payments, with detrimental effects on the poorest and most in need. Evidence from high income countries shows that depression is associated with increased healthcare utilization, which may lead to increased OOP expenditures, placing greater stress on families. To inform policies for integrating mental healthcare into UHC in LMIC, we must understand healthcare utilization and OOP expenditure patterns in people with depression. We examined associations between symptoms of depression and frequency and type of healthcare utilization and OOP expenditure among adults in Chitwan District, Nepal.

**Methods:**

We analysed data from a population-based survey of 2040 adults in 2013, who completed the PHQ-9 screening tool for depression and answered questions about healthcare utilization. We examined associations between increasing PHQ-9 score and healthcare utilization frequency and OOP expenditure using negative binomial regression. We also compared utilization of specific outpatient service providers and their related costs among adults with and without probable depression, determined by a PHQ-9 score of 10 or more.

**Results:**

We classified 80 (3.6%) participants with probable depression, 70.9% of whom used some form of healthcare in the past year compared to 43.9% of people without probable depression. Mean annual OOP healthcare expenditures were $118 USD in people with probable depression, compared to $110 USD in people without. With each unit increase in PHQ-9 score, there was a 14% increase in total healthcare visits (95% CI 7–22%, *p* < 0.0001) and $9 USD increase in OOP expenditures (95% CI $2–$17; *p* < 0.0001). People with depression sought most healthcare from pharmacists (30.1%) but reported the greatest expenditure on specialist doctors ($36 USD).

**Conclusions:**

In this population-based sample from Central Nepal, we identified dose-dependent increases in healthcare utilization and OOP expenditure with increasing PHQ-9 scores. Future studies should evaluate whether provision of mental health services as an integrated component of UHC can improve overall health and reduce healthcare utilisation and expenditure, thereby alleviating financial pressures on families.

## Background

In an international pursuit to reduce global poverty and improve population health, the world’s governments have committed, in the Sustainable Development Goals, to achieve universal health coverage (UHC), including financial risk protection by 2030 [1]. A critical decision in the progressive realisation of UHC is which health services to include in essential packages of care. There is evidence that mental health interventions can be cost-effective [2, 3], and several prominent mental health advocates have recommended their inclusion in UHC [4–7]. Besides being a major contributor to the burden of disability worldwide [8], much of which falls on low- and middle-income countries (LMIC) [9], there are also strong economic arguments for the inclusion of basic mental health care in UHC. The global economic burden of mental disorders comes to $2.5 trillion USD per year, projected to double by 2030, of which two thirds arises from lost earnings and one third directly from healthcare expenditure [2].

To inform national policies for achieving UHC, and decisions around which interventions to prioritise in low-resource settings, we need to understand patterns of healthcare utilization and out-of-pocket payments (OOP). There is evidence from North America and Europe that healthcare expenditure is more than twice as high in people with depression than those without [10–14] suggesting that this group places a disproportionate burden on health systems, whose needs are important to consider when planning UHC. However, it is unknown whether this finding is generalisable to other countries with vastly different health systems. In LMIC, where healthcare is predominantly financed by OOP payments [15], any increased utilization can lead to impoverishment, itself a major risk factor for depression [16], and can result in the poorest individuals foregoing necessary care [17].

Nepal is a low-income fragile state, where prolonged conflict, trauma and natural disasters have contributed to a weakened health system in which 60% of total healthcare expenditure is privately funded, of which 80% is OOP [18]. The Nepalese government has made significant efforts to improve UHC in the last 5 years, with social health insurance now available in 42 out of 77 of its districts [19]. However, uptake remains low, with only 6% of the population signing up to the scheme [19], and specialist mental health services remain unavailable for the majority of individuals with mental disorders [20]. At present there is no data from Nepal on healthcare utilization by people with depression, in order to inform policies around including depression treatment care in UHC to address this group’s needs and reduce OOP spending. While there is some evidence that OOP expenditures increase among older people with depression in southern India [21], the available evidence from elsewhere in LMIC shows that community healthcare utilization by people with depression varies considerably between countries [22].

Recent evidence from the Programme for Improving Mental Health CarE (PRIME) [23] demonstrates the feasibility [24, 25] and effectiveness of integrating mental health services into primary healthcare settings in Nepal, based on the WHO Mental Health Gap Action Programme (mhGAP) [26]. Modelled estimates suggest that this kind of integration could be scaled-up nationally for an investment of $5.55 US dollars per capita, spread over 7 years [27]. The PRIME approach provides a potential model upon which to base integrated services if mental health care were included in UHC. Understanding patterns of healthcare utilization and OOP expenditure by people with depression can help us to predict the likely financial and health service impacts of implementing this policy in Nepal.

In this secondary analysis of data from the PRIME study, we set out to examine associations between increasing symptoms of depression and healthcare utilization and related OOP expenditure among adults in one district in Central Nepal. In our definition of healthcare utilization, we included hospitalizations and use of any outpatient healthcare services. These included both generalist and specialist doctors, mental health specialists, pharmacists and providers of traditional and complementary medicine. Our secondary objective was to compare utilization of different outpatient service providers among adults with and without probable depression and to estimate the mean expenditure associated with each provider type.

## Methods

### Setting

This study was carried out in Chitwan district in central Nepal, where some mental health services were already in existence at the time of study, as described previously [28, 29]. In previous studies, approximately 41% of people reported symptoms of depression in the north-western mountains [30], compared to 3% in this sample from the central plains [31], of whom only 8.1% accessed treatment [31]. Despite Chitwan having adopted the National Mental Health Policy in 1996, there were only 2 psychiatrists serving its population of over half a million [32] at the time of the study.

### Sampling and participants

The rationale, study design and data collection procedures for the PRIME study have been detailed previously [31]. Briefly, the PRIME study included a population-based survey of adults in Chitwan District. Using household and population data from Village Development Committees (VDCs; similar to municipalities, with community participation in their administration) in Chitwan, the lead investigator randomly selected houses from each VDC, where field workers enumerated the adults in the household and a family member randomly selected 1 adult from a series of concealed papers for recruitment into the study. Eligibility criteria included age of 18 years or over, residency in Chitwan, fluency in the Nepali language and willingness and ability to provide informed consent. Between May and August 2013, 99% of eligible adults provided informed consent and 2040 adults participated in the study.

All PRIME participants answered questions about demographic characteristics and received screening for probable depression (described below) and alcohol-use disorder in Part 1 of a two-part survey. To facilitate the analysis of secondary research questions without overburdening research staff and participants, questions about household economic status, healthcare utilization and OOP expenditure were only included in Part 2 of the survey (also described below), which was limited to a sub-sample of 479 participants. This sub-sample included all participants reporting symptoms of depression (acute or chronic) (213), alcohol-use disorder (78) or both (18) and a random 10% sample of remaining screen negative participants (170) as shown in Additional File 1. The decision to include 10% of remaining participants was based on an estimated 10% prevalence of alcohol use disorder or depression, thereby enabling comparisons of equal numbers of screen positive and screen negative participants.

The questionnaire was orally administered by a trained fieldworker in the Nepali language and responses were collected on a questionnaire application, which was programmed onto an Android mobile device.

### Measures

#### Depression status

We screened participants for depression with the 9-item Patient Health Questionnaire (PHQ-9) [33]. The PHQ-9 grades self-reported symptoms of depression in the previous 2 weeks and severity is determined by increasing scores. We interpreted a score of 10 or more to reflect probable depression, which in a Nepali validation study had a sensitivity of 0.94 and specificity of 0.80 [34]. In this sample, the PHQ-9 had a Cronbach’s alpha of 0.79 [31].

#### Household economic status

Household economic status was evaluated on the basis of household assets (such as water supply, sanitation facilities, power supply, radios, televisions, mobile phones, cooking fuel, a separate kitchen and type of flooring) using an asset score, which was developed specifically for use in Nepal for the purposes of this study and described further in Additional File 2. Employing the methods described by Vyas and Kumaranayake [35], we used principal component analysis to generate a relative wealth index from the asset score, which we subsequently categorized into thirds to reflect low, average and high wealth categories.

#### Healthcare utilization

We assessed inpatient and outpatient healthcare utilization and OOP healthcare expenditure using a version of the Client Socio-demographic and Service Receipt Inventory [36], which has been adapted for use in LMIC [37]. To measure inpatient healthcare utilization, we asked participants to report the number of times they had been admitted to hospital in the previous 12 months, if at all. We then asked participants to report the number of times, if any, they had visited outpatient services for any health problem (including but not exclusively for depression) over the previous 3 months including seven types of healthcare providers: traditional healers, community workers, nurse/midwives, pharmacists, general doctors, specialist doctors, and psychiatrists and other mental health workers. In order to make comparisons between inpatient and outpatient healthcare utilization and to calculate an estimate of total utilization, we standardized the number of visits in 3 months to reflect annual outpatient healthcare utilization as reported in similar analyses [10, 17, 38]. We also recorded data on the presenting health complaint.

#### Healthcare expenditure

For each inpatient hospital admission reported in the past 12 months, we asked participants to report all individual payments for hospital fees, medicines, laboratory tests and other investigations (including scans), and transportation incurred both personally and by friends and family. We used the sum of all these payments to estimate the annual inpatient OOP expenditure. The inclusion of payments for transportation to and from health facilities in the definition of OOP expenditures is consistent with other OOP cost studies from LMIC [39].

For each episode of outpatient healthcare utilization over 3 months, we also asked participants to report all individual OOP payments for consultations with western biomedical practitioners or providers of traditional and complementary medicine as well as return transportation. We also standardised these payments to reflect annual outpatient OOP expenditure. Finally, we summed the annual inpatient and outpatient OOP expenditures to estimate the total annual OOP expenditure. We did not include opportunity costs or indirect costs such as lost productivity and all costs were defined from the user’s perspective. All expenditures were reported in Nepali Rupees and converted to US dollars according to the exchange rate at the end of data collection (1 USD:96.997 Nepali rupee on 02 Aug 2013). We observed one implausible outlier for outpatient OOP expenditure and replaced it with the sample’s mean outpatient OOP cost.

### Statistical analysis

First, we report participants’ demographic [40] characteristics, PHQ-9 scores, frequency of healthcare utilization and mean OOP expenditures, stratified by depression screening status. Categorical measures are summarized as proportions, age as means and standard deviations and PHQ-9 scores and healthcare utilization (whose distribution was skewed), as medians and interquartile ranges. We present mean annual OOP expenditures, overall and by depression screening status, employing bootstrapping methods with 1000 replications to estimate standard errors. This method enables estimation of mean expenditures required for health budgeting [41], whilst accounting for the skewed distribution typical of cost data [42, 43] and has been used with survey data of this nature previously [44]. One participant who had missing age was imputed to 39.8 years, the mean value for the overall sample. Second, we assessed the relative changes in annual inpatient, outpatient and total healthcare utilization (by number of visits) for each unit increase in PHQ-9 score in all participants. Given the skew in the distribution of healthcare utilization, which precluded use of linear regression, we used negative binomial regression. Third, we estimated the relative changes in inpatient, outpatient and total OOP healthcare expenditure for each unit increase in PHQ-9 score using bootstrapped linear regression models. We adjusted for age, sex and relative wealth index in all models. We used the relative wealth index as a proxy for household economic status and specifically for access to basic necessities, which has been shown in Nepal to predict healthcare utilization [15]. In exploratory analyses, we also adjusted for education and occupation as potential confounders of the association between depression and healthcare utilization but omitted these from the primary model to avoid collinearity. We also checked for interactions between PHQ-9 score and relative wealth group. Finally, we report utilization and mean OOP expenditures for each outpatient provider type, stratified by probable depression status.

For each step, survey-adjusted methods were used to account for the complex sampling design and sampling probability weights [45]. Data were analysed using Stata version 14.2 [40]. Ethical approval was obtained from the Nepal Health Research Council (Kathmandu, Nepal), the World Health Organization (Geneva, Switzerland) and the London School of Hygiene and Tropical Medicine.

## Results

### Demographic characteristics, PHQ-9 scores, healthcare utilization, and OOP expenditures

Table 1 displays the demographic characteristics, PHQ-9 scores, health care utilization and expenditure of community survey participants. The mean age of all 2040 participants was 39.8 years (SD 15.2), of whom 59.8% were female and 54.5% had secondary level education or higher. The median PHQ-9 score was 2 with a range 0 to 24 and the point prevalence of probable depression (PHQ-9 ≥ 10) was 3.6%. Probable depression was more common in participants with the lowest (6.9%) compared to the highest (2.0%) educational attainment), unskilled workers (4.2%) compared to business employees (1.9%) and those among the poorest (6.0%) compared to the wealthiest (3.1%) third. Of all 479 adults who answered questions about healthcare utilization, 233 had not used any healthcare, whereas 18 had been admitted to hospital, 197 had utilised outpatient services and 31 had used both. People with probable depression were twice as likely to be admitted to hospital (15.8%) as people without (7.8%) and were also more likely to visit outpatient services (69.7%) than people without (39.8%). Participants reported seeking outpatient healthcare for a wide range of ailments at the first visit. For individuals with probable depression this was most commonly for signs and symptoms of infection (20.7%), followed by depression, anxiety or sleep disturbance (14.0%), other chronic conditions (12.8%), joint pains (9.5%), gastrointestinal symptoms (most commonly heartburn) (9.2%), injuries (8.0%) or routine check-ups (7.9%) with all other categories under 5%. Among healthcare users without depression, most used healthcare for joint pains (22.1%), followed by acute conditions such as allergy (17.1%), gastrointestinal symptoms (13.9%), infections (9.9%) and other chronic conditions (8.1%). 88% of participants who utilised healthcare incurred some OOP expenditure and the mean total OOP expenditure was $118 USD/year (SD 265) in adults with probable depression and $110 USD (SD 879) in people without depression.

**Table 1:** PHQ-9 scores, sociodemographic and healthcare-related characteristics of adults in Chitwan District, Nepal, 2013

	N	Overall (%)	PHQ-9 Negative *N* = 1960 (96.41%)	PHQ-9 positive *N* = 80 (3.56%)
PHQ-9 Scores	**2040**			
Range		0–24	0–9	10–24
Median (IQR)		2 (0–4)	2 (0–4)	11 (10–14)
Demographic Characteristics	**2040**			
Mean age (SD)		39.77 (15.23)	39.54 (15.06)	45.87 (18.49)
Female	1336	59.75%	96.44%	3.56%
Male	704	40.25%	96.41%	3.59%
Educational Status				
Sub primary/illiterate	275	12.83	93.06%	6.94%
Primary/literate	686	32.68	95.17%	4.82%
Secondary or higher	1079	54.49	97.96%	2.04%
Occupation				
Agriculture, Labour or Service	1409	66.40	95.73%	4.23%
Business and Service	302	15.46	98.15%	1.85%
Unemployed	94	4.88	97.01%	2.93%
Student	161	9.99	97.73%	2.27%
Other	74	3.26	97.22%	2.78%
Relative wealth index^a^				
Low	155	22.27	94.00%	6.00%
Average	162	32.57	96.74%	3.26%
High	161	45.16	96.92%	3.07%
Healthcare Utilization^a^	**479**		**399**	**80**
Inpatient	49			
Percentage with any admissions		8.02	7.82	15.83
Median annual number of visits (IQR)		0 (0–0)	0 (0–0)	0 (0–0)
Outpatient	228			
Percentage with any outpatient visits		39.99	38.83	69.70
Median annual number of visits (IQR)		0 (0–8)	0 (0–4)	4 (0–8)
Total	246			
Percentage with any healthcare utilization		44.93	43.91	70.85
Median annual number of visits (IQR)		1 (0–8)	0 (0–5)	4 (0–8)
Healthcare Expenditure^a^	**479**			
Inpatient	47			
Percentage with any expenditure		99.06	99.19	97.46
Mean annual OOP expenditure in USD (SD)		65.94 (766.01)	68.12 (820.59)	53.95 (194.63)
Outpatient	191			
Percentage with any expenditure		85.58	85.60	88.65
Mean annual OOP expenditure in USD (SD)		44.85 (130.81)	41.44 (124.81)	63.71 (161.17)
Total	212			
Percentage with any expenditure		87.86	87.44	97.43
Mean annual OOP expenditure in USD (SD)		110.80 (822.78)	109.55 (878.97)	117.66 (265.46)

Demographic characteristics are presented as row percentages, while healthcare utilization and expenditure are presented as column percentages

Age and OOP healthcare expenditures (US Dollars) are presented as means and standard deviations (SD), using bootstrapping techniques with 1000 bootstrap samples to estimate the SD for OOP expenditures

PHQ-9 scores and number of healthcare visits are presented as medians and interquartile ranges (IQR)

The percentage with any expenditure is calculated as a proportion of those who utilised inpatient or outpatient care respectively

aRelative wealth index, healthcare utilization and healthcare expenditure were assessed in 479 of the total 2040 participants who answered part 2 of the survey and all percentages and means are adjusted for sampling-probability weights

### Healthcare utilization and expenditures and depression severity

The associations between PHQ-9 score and healthcare utilization and expenditure are displayed in Table 2. In univariate analyses, each unit increase in PHQ-9 score was associated with a 14% increase in outpatient healthcare consultations, a 14% increase in total healthcare consultations and a weaker 7% increase in inpatient admissions. After adjustment for age, sex and relative wealth the estimates for outpatient (15% CI 6–23%, *P* < 0.0001), total (15% CI 7–22%, *P* < 0.0001), and inpatient (7% (95% CI -6-22%, *P* = 0.314) healthcare utilization were materially unchanged.

**Table 2:** Associations between PHQ-9 scores and annual healthcare utilization and OOP expenditure among adults in Chitwan district, Nepal, 2013

		Utilization (Number of visits)		OOP Expenditure (US dollars)	
		IRR (95% CI)	***P*** value	β (95% CI)	***P*** value
Inpatient Admissions *N* = 479	**Univariate**	**1.08 (0.94–1.25)**	**0.280**	**3.63 (−0.99–8.26)**	**0.124**
	**Multivariate**	**1.07 (0.94–1.22)**	**0.314**	**4.00 (−1.84–9.84)**	**0.179**
	Age	*1.00 (0.94–1.22)*	*0.981*	*4.37 (−3.63–13.49)*	*0.284*
	Female	*0.70 (0.32–1.54)*	*0.378*	*−83.84 (−211.31–43.62)*	*0.197*
	Relative wealth index	*0.96 (0.71–1.30)*	*0.794*	*26.92 (−19.08–72.92)*	*0.251*
Outpatient Consultations *N* = 479	**Univariate**	**1.14 (1.06–1.23)**	**< 0.0001**	**5.52 (2.36–8.67)**	**0.001**
	**Multivariate**	**1.14 (1.06–1.23)**	**< 0.0001**	**5.36 (2.26–8.47)**	**0.001**
	Age	*1.00 (0.98–1.01)*	*0.549*	*0.62 (−0.22–1.45)*	*0.150*
	Female	*1.07 (0.66–1.71)*	*0.786*	*17.26 (−7.58–42.09)*	*0.173*
	Relative wealth index	*1.00 (0.66–1.71)*	*0.986*	*6.49 (0.18–13.11))*	*0.044*
Total Healthcare *N* = 479	**Univariate**	**1.14 (1.07–1.22)**	**< 0.0001**	**9.15 (3.12–15.18)**	**0.003**
	**Multivariate**	**1.14 (1.07, 1.22)**	**< 0.0001**	**9.37 (2.15–16.58)**	**0.011**
	Age	*1.00 (0.98–1.01)*	*0.563*	*4.99 (−3.52–13.49)*	*0.251*
	Female	*1.05 (0.67–1.65)*	*0.817*	*−66.59 (−204.87–71.70)*	*0.345*
	Relative wealth index	*1.00 (0.88–1.13)*	*0.982*	*33.57 (−16.17–83.31)*	*0.186*

Healthcare utilization and OOP Expenditure were estimated using negative binomial regression and bootstrapped linear regression to model incidence rate ratios (IRR), and β coefficients respectively along with and 95% confidence intervals

Both IRR and β coefficients were adjusted by survey-sampling weights and multivariate analyses were further adjusted for sex, gender and relative wealth index

Estimates reflect changes associated with a one unit increase in PHQ-9 score after adjustment for age, gender and relative wealth index

For each increment in PHQ-9 score, OOP expenditure on outpatient and total healthcare increased by $5.52 USD and $9.15 USD per year respectively. After adjustment for age, sex and relative wealth, these OOP expenditures were unchanged for outpatient ($5.36 USD (95% CI 2.36–8.47)) and total ($9.37 USD (95% CI 2.15–16.58) healthcare utilization (Table 2).

There was only weak evidence for differences in inpatient expenditures. None of these reported associations were modified by relative wealth index during initial inspection of the data. Exploratory analyses adjusting additionally for educational attainment and occupation yielded similar associations between depression and total healthcare utilization (17% (95% CI 9–25%) (including outpatient 17% (95% CI 9–27%); and inpatient 12% (− 3–29%)) and total OOP healthcare expenditures ($9.84 (1.39–18.30) (including $5.35 (95% CI 2.30–8.40) for outpatient and $4.49 (− 2.55–11.54) for inpatient). Similarly, the reported associations between depression and healthcare utilization were similar after excluding 21 people who used healthcare for chronic health problems.

### Healthcare utilization by provider type

**Fig. 1:**
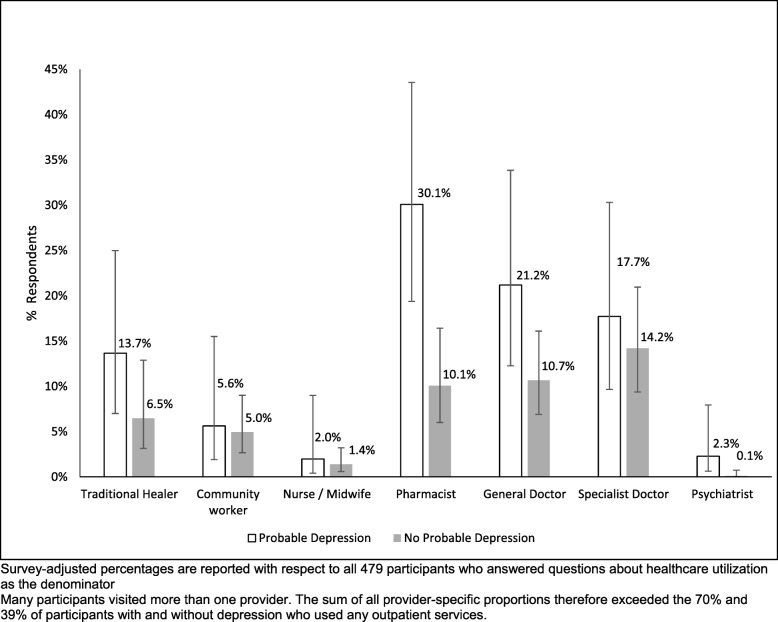
Outpatient Provider-Specific Healthcare utilization by probable depression status among adults in Chitwan District, Nepal, 2013

Figure 1 displays the proportion of people with and without probable depression who used each type of outpatient healthcare. For each provider, the proportion of people who reported any outpatient consultations in the past 3 months was greater among those with probable depression than those without. Of note, people with depression most commonly sought treatment from pharmacists (30.1%), who they were 3 times more likely to visit than people without probable depression. General doctors were the second most common source of treatment seeking in the depressed group (21.2%). In comparison, people without depression consulted (non-mental health) specialist doctors (17.7%) most frequently, whilst few people overall visited traditional healers (6.7%) or psychiatrists (0.2%).

### Provider-specific healthcare expenditure

**Fig. 2:**
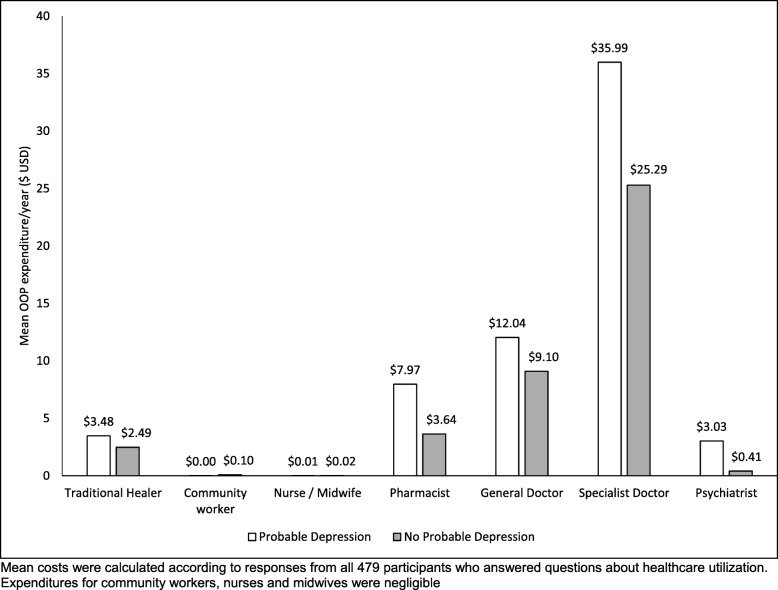
Outpatient sector-specific mean OOP healthcare expenditures (US Dollars/yr) by probable depression status

Participants with probable depression incurred higher annual OOP expenditures for each provider type on average than participants without, as shown in Fig. 2. In contrast to utilization patterns, these expenditures were highest for visits to specialist doctors ($35.99 USD (SD 15.12)), followed by general doctors ($12.44 USD (SD 6.11)) and pharmacists ($7.97 USD (SD 3.28)), compared to $3.48 USD (SD 14.21) for traditional healers and $3.03 USD (SD 24.26) for psychiatrists.

## Discussion

### Principal findings

Our results demonstrate that people with probable depression in Central Nepal used more healthcare and incurred greater OOP expenditures than people without depression, and that both utilization and OOP expenditure increased significantly with increasing depression screening scores. People with probable depression incurred total mean expenditures of $118 (SD 29) USD/year OOP on healthcare, which is likely to represent a substantial proportion of household budgets considering that median annual income for adults with depression in this area has been estimated to be $501 [17]. *The association between higher PHQ-9 score and total healthcare utilization and OOP costs was attributable to outpatient rather than inpatient service use, and was not modified by relative wealth.*

Our findings also show that when seeking healthcare, individuals with probable depression most often sought care from pharmacists, with very few consulting mental health specialists for healthcare. Despite their more frequent use, annual pharmacist costs were much lower than for consultations with specialist doctors ($7.97 USD/year compared to $36.99 USD/year) and general doctors ($12.04 USD/year)*.*

### Strengths and limitations

This is the only population-based study *that we are aware of from Nepal or any other low-income country* to simultaneously evaluate dose-dependent associations between depression and healthcare utilization and OOP expenditure and to examine utilization of different service providers. *We also* minimised the risk of misclassification bias because we validated the PHQ-9 in this setting and used the total PHQ-9 score as a continuous variable for most statistical analyses.

However, our findings should be interpreted in light of some limitations. Firstly, Due to the cross-sectional design, (and because the PHQ-9 refers to symptoms of depression in the previous 2 weeks, whereas the CSRI records healthcare utilization and expenditure for the previous 3 to 12 months), it is difficult to infer causality or determine the true temporal relationship between depression and healthcare utilization. However, depression typically adopts a chronic relapsing and remitting course and it is likely that these symptoms preceded the 2 week recall period.” Secondly, in the absence of reliable health service records, we measured utilization and expenditure by self-report, which may be subject to recall bias, although we restricted the recall period for outpatient utilization to 3 months to minimise this. Conversely, by extrapolating outpatient healthcare utilization from 3 months to 1 year, we may have introduced infrequency bias due to seasonal fluctuations, which could have led us to over or underestimate these associations. Thirdly, we cannot rule out unmeasured confounding from urban or rural residence, which are known to influence healthcare access in Nepal [46, 47] *or* physical comorbidities, as neither were recorded in this study. However, only 13% of participants with depression who used healthcare reported doing so for chronic health problems and previous studies that have adjusted for comorbidities have still found residual associations [22]. Fourth, these findings may not be generalizable to other regions in Nepal, although we used a large, population-based sample, in which secondary level educational attainment was fairly representative of national estimates, suggesting that this was not an obvious source of selection bias [48]. Finally, this was a secondary data analysis designed to explore healthcare utilization in people with depression in Nepal and we could not therefore perform sample size calculations a priori. We have also not performed retrospective power calculations due to the nature of the exposures and outcomes and the inaccuracies associated with these types of calculations [49–51]. However, our results may still be subject to errors of either sign (Type S) or magnitude (Type M), which we are unable to account for [49]. Our findings are therefore more exploratory than confirmatory and adequately powered prospective studies are now necessary to confirm these associations and establish the direction of causation and the underlying mechanisms.

### Comparison with previous literature

The pattern of increased healthcare consumption by people with probable depression in Nepal reported here is consistent with observations from high-income countries, where the greatest direct healthcare costs are also attributable to general healthcare rather than specialist mental healthcare utilization [12]. Our finding that excess expenditure by people with depression was largely accounted for by outpatient service use is consistent with findings from India [21, 52], Brazil [53] and other middle-income countries [22], which demonstrate 14 to 36% increases in community healthcare utilization among people with depression, and suggest that depression is a risk factor for catastrophic health expenditure [37]. This is the first study that we know of to independently report hospital admission frequency and OOP healthcare expenditures in people with symptoms of depression in LMIC, although a study from Canada also found no significant increases in health service related costs of admissions [12].

Whether people with depression seek more help from pharmacists in LMIC has not been studied before, but in Brazil it has been reported that depressed individuals use more medications than the general population [53]. It is important to note that the role of pharmacists in Nepal is generally limited to the provision of over the counter drugs rather than diagnoses or prescriptions. As reported elsewhere in Nepal [46], we detected minimal utilization of traditional healers for overall healthcare needs. This may be due to increased stigmatization of the use of traditional healers [54] and a general preference for ‘pill-based’ treatments in South Asia [55]. Conversely, only 2% of people with depression who utilized outpatient services were seen by a psychiatrist, which is consistent with previous literature and the shortage of mental health specialists in the area [32]. *Our findings are therefore more exploratory than confirmatory and adequately powered prospective studies are now necessary to confirm these associations and establish the direction of causation and the underlying mechanisms.*

### Mechanisms and implications

**Fig. 3:**
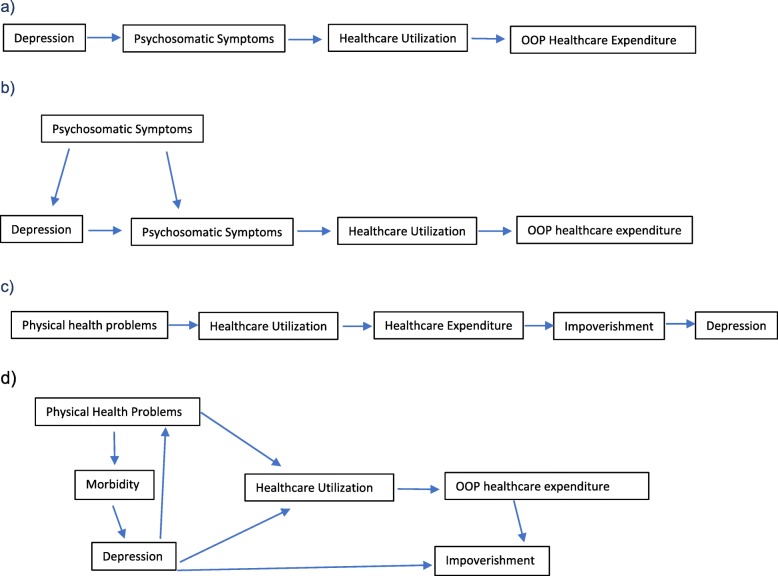
Schematic Representation of Potential Causal Pathways Between Depression and Healthcare Utilization and OOP Expenditure

Given the limitations of the data described above, there are multiple potential explanations of the association between depression symptoms and healthcare utilization and expenditure, which are not necessarily mutually exclusive: (1) The first explanation is that depression leads to increased utilization of services through somatization – that is, the manifestation of psychological distress as physical symptoms – which has been demonstrated previously [56] (Fig. 3a). (2) Alternatively, depression may lead to increased healthcare utilization because of its association with physical comorbidities, which are both a risk factor for and a consequence of depression [57–59] (Fig. 3b). (3) Finally, OOP healthcare expenditure can result in poverty [38], which in turn is a major risk factor for depression [16], raising the possibility that the causal pathway acts in the opposite direction (Fig. 3c).

There is evidence to support the plausibility of each of these pathways. In support of the first and second hypotheses, which posit a causal role of depression, associations have been shown prospectively between depression and health service utilization in Canada [12] and the United States [60]. Further, somatization is a common presentation of depression in South Asia [61–65] and particularly in Nepal [66], which could explain the increased demand for physical rather than mental healthcare [56] and might possibly be consistent with the 20% or so who sought care for gastrointestinal symptoms or joint pains (although somatization is a diagnosis of exclusion and we cannot assume that these are not indicative of real underlying pathology). Previous analyses have also shown that only 8.1% of people with probable depression in this sample reported actively seeking treatment specifically for depression symptoms [67], which is far lower than the 71% who sought any healthcare at all. This may relate to the lack of effective detection and treatment of depression in primary care [68, 69], and possibly to low perceived need for intervention for psychological symptoms [70].

At the same time, a growing evidence base also demonstrates that depression increases the risk of a variety of physical health problems [71–74], which could mediate the relationship between depression and healthcare utilization. Cross-sectional evidence from Nepal supports an association between depression and chronic comorbidities [75, 76]. Nonetheless, studies that have controlled for these comorbidities have still found a residual association between depression and increased healthcare utilization, suggesting that depression also exerts an independent effect on treatment-seeking [22].

Recent evidence shows that primary care workers can be trained [24] to improve detection of depression and deliver mhGAP-based interventions [26] (including psychoeducation and medication) to effectively treat depression and reduce disability in Nepal [25]*.* If depression is the primary driver of increased healthcare utilization among this group (either directly, by somatization, or indirectly, by impacting on general health) then we would expect the roll-out of such services to reduce excess healthcare use and associated OOP expenditure. Elsewhere, the integration of mental health services into primary care in Andhra Pradesh in India [39] has been shown to substantially reduce OOP healthcare expenditures. In Nepal, the estimated combined costs of scaling-up services for psychosis, depression and epilepsy are less than $1.30 USD/capita/year [27]. In contrast to the additional $9 USD/year OOP incurred by people in this study for each increment in PHQ-9 score, this is a relatively small investment, which might minimise financial impoverishment, whilst reducing symptoms of depression and hopefully break the link between the two.

However, the notion that OOP healthcare expenditures lead to depression (Fig. 3c) also finds some empirical support. Healthcare expenditure represents an exceptionally large share of total household expenditure in Nepal [15] and therefore poses a risk of catastrophic expenditure, impoverishment and widened inequalities [15], which are significant determinants of depression [16]. In support of this, we found that the poorest individuals were most likely to suffer from depression but did not appear to forego care, which we would expect to cause further impoverishment. We also observed that people with depression commonly presented with signs and symptoms of infection, which we also expect to be more common in areas of deprivation. Thus, the lack of financial protection for people with depression is likely to reinforce a vicious cycle of further healthcare utilization and greater impoverishment*.* According to this model, introducing financial protections would reduce depression prevalence. Supporting the uptake of social health insurance schemes in Nepal could therefore break the cycle between the impoverishing effects of healthcare utilization and the associated risks of depression. In support of this hypothesis, studies from the United States show that introduction of Medicaid led to 9% reductions in depression prevalence and near elimination of catastrophic expenditure [77].

Given previous evidence of the inter-relationships between poverty and financial shocks, depression, and physical health, it seems probable that the observed association between depression and healthcare utilization and expenditure results from a combination of these pathways Fig. 3d). Indeed, the idea that physical health, mental health and poverty interact synergistically through multiple complex pathways underlies syndemics theory [78–80]. Further research would be necessary to empirically confirm the relative contribution of each of these mechanisms in the Nepali context, but a combination of increased financial protection for healthcare and the provision of effective depression treatment might reasonably be recommended to break this cycle.

In terms of how to provide depression treatment as part of UHC, the low frequency of hospital admissions support proposals to integrate mental health services into primary rather than secondary care in LMIC. The low cost and frequent consultation of pharmacists, relative to general and specialist doctors, is also relevant to policy as it indicates an additional cadre of healthcare providers who are in frequent contact with the target population. Evidence from PRIME demonstrates that task-sharing by training and supervising non-specialist health workers can improve depression detection rates [69] and mhGAP-based interventions for depression in Nepal have been found to be most effective when they were supplemented with psychosocial treatments delivered in the community [24]. Our findings suggest that enlisting pharmacists to identify potential depression patients and refer them to community counsellors to receive appropriate treatments such as the Healthy Activity Program [81], might be a fruitful strategy to both reduce the provision of unnecessary medications (and therefore OOP costs) and ultimately provide both mental and physical health benefits [25].

### Unanswered questions and future research

This study contributes to the evidence base on healthcare utilization and expenditure by demonstrating that, in Nepal, adults with probable depression use a disproportionate amount of healthcare and incur increased healthcare expenditures. Future studies should also investigate whether providing mhGAP-based interventions for depression mitigates excess utilization of health services by people with depression, to inform their inclusion in social health insurance packages of care and to prevent impoverishment in this group. Modelling studies have estimated that strengthening primary health care in LMIC to diagnose and treat depression could result in productivity gains as high as $4 USD for every $1 USD invested [82] and the real-world effect of such an approach should therefore be evaluated. Equally, the impact of expanding financial protection for depression as part of UHC should also be evaluated in LMIC. Finally, the feasibility of training pharmacists in Nepal to recognise depression and signpost individuals with depression to appropriate care should be investigated.

## Conclusion

Our findings show that people with symptoms of depression in central Nepal utilise more healthcare and incur greater OOP costs compared to people without depression. This economic burden is accounted for by frequent visits to pharmacists and costly visits to general and specialist doctors, with minimal use of specialist mental health care. These findings reinforce the emerging consensus that policy-makers in low-income countries must improve universal access to cost-effective treatments for depression through integration of mental healthcare services into primary healthcare, as well as expanding financial protection as part of universal health coverage initiatives, to reduce the financial burden of depression on patients. Engaging pharmacists and primary care doctors as key stakeholders may help to improve case detection and access to appropriate evidence-based services, which can be delivered in the community. Future research should investigate the impact of financial protection on depression, and of depression treatment on healthcare utilization and expenditure.

## Supplementary information

**Additional file 1.** Sampling Procedure for the Community Surveys for the Programme for Improving Mental Health CarE (PRIME) In Nepal.

**Additional file 2.** Relative Wealth Asset Index to Determine Household Economic Status in the Programme for Improving Mental Health CarE (PRIME) in Nepal.

## Data Availability

The datasets analysed during the current study are not publicly available immediately due to organisational policy and ethical considerations. In the consent forms, we did not ask for consent from participants for their data to be made publicly available. However, we have set up a system outlined in our publication policy where interested researchers can apply for access to the data via the PRIME consortium Expression of Interest form which is available here: http://www.prime.uct.ac.za/ contact-us. The data access committee is made of the members of the PRIME management group, led ﻿by the PRIME CEO, Prof Crick Lund at the University of Cape Town. All requests for data will go through the PRIME Expression of Interest form which is available in PRIME website http://www.prime.uct.ac.za/contact-us. Anyone who is interested in collaboration or using PRIME data can fill in the form and submit it. As described in our publication policy, the request is vetted by the PRIME Management Team and the PRIME consortium. Additional details can be obtained from Erica Breuer, PRIME project manager (Erica. breuer@uct.ac.za).

## Abbreviations

IQR: Interquartile range
IRR: Incident rate ratio
LMIC: Low- and middle-income countries
MhGAP: Mental Health Gap Action Programme
OOP: Out-of-pocket expenditures
PHQ9: Patient Health Questionnaire
PRIME: Programme for Improving Mental Health CarE
SD: Standard deviation
UHC: Universal Health Coverage
USD: United States Dollars
VDC: Village Development Committee
