# Supplementary material for: Healthcare utilization and out-of-pocket expenditures associated with depression in adults: a cross-sectional analysis in Nepal
Source: BMC Health Serv Res. 2020 Mar 25;20:250. doi: 10.1186/s12913-020-05094-9 (PMC7093962; doi:10.1186/s12913-020-05094-9)
Supplement: Supplementary file 1 — Additional file 1. Sampling Procedure for the Community Surveys for the Programme for Improving Mental Health CarE (PRIME) In Nepal. [file 12913_2020_5094_MOESM1_ESM.docx]

## Sampling Procedure for the Community Surveys for the Programme for Improving Mental Health CarE (PRIME) In Nepal

α Screen-positives for depression also included individuals who gave affirmative responses to the question:

*‘Apart from these past two weeks, during the past 12 months, did you have other episodes of two weeks or more when you felt depressed or uninterested in most things, and had most of the problems we just talked about?’*

β AUD: Alcohol use disorder – determined by a score of ≥16 on the Alcohol-use Disorders Identification Tool
