# Supplementary material for: Healthcare utilization and out-of-pocket expenditures associated with depression in adults: a cross-sectional analysis in Nepal
Source: BMC Health Serv Res. 2020 Mar 25;20:250. doi: 10.1186/s12913-020-05094-9 (PMC7093962; doi:10.1186/s12913-020-05094-9)
Supplement: Supplementary file 2 — Additional file 2. Relative Wealth Asset Index to Determine Household Economic Status in the Programme for Improving Mental Health CarE (PRIME) in Nepal. [file 12913_2020_5094_MOESM2_ESM.docx]

## Relative Wealth Asset Index to Determine Household Economic Status in the Programme for Improving Mental Health CarE (PRIME) in Nepal

﻿**Excerpted from:** Jordans M, Rathod S, Fekadu A, Medhin G, Kigozi F, Kohrt B, et al. Suicidal ideation and behaviour among community and health care seeking populations in five low- and middle-income countries: a cross-sectional study. Epidemiol Psychiatr Sci. 2017;1–10.

“To create country-specific relative economic scores in Nepal and South Africa, we used principle components analysis and included a range of locally-relevant assets and dummy-coded housing characteristics. The sum of weights from the first factor from principle components analysis is a Z-score on a Normal distribution, which corresponds to each participant’s relative wealth in comparison to the other respondents in the country dataset.^1^ We created tertiles of these scores to classify respondents as being of lower, average, or higher economic status, relative to their neighbours.”

### Assets Included in the Relative Wealth Asset Score:

Responses to the following questions, were dichotomized as yes or no before using principle components analysis to generate the index.

1. What is the main source of drinking water for members of your household?

Spring well

Tap water

Borehole

Rainwater

River

Tube-well

1. What kind of toilet facility do members of your household usually use?

Flush toilet

Pit latrine

Bucket toilet

Bush/field

1. Does your household have electricity?
2. Does your household have a radio?
3. Does your household have a television?
4. Does your household have a mobile telephone?
5. What type of fuel does your household mainly use for cooking?

Electricity

Gas

Kerosene

Wood

Animal dung

Bio-gas

No food cooked in household

1. Do you have a separate room which is used as a kitchen
2. Floor type

Earth Sand

Wood/bamboo

Finished floor

Rudimentary

### References:

1. Vyas S, Kumaranayake L. Constructing socio-economic status indices: How to use principal components analysis. Health Policy Plan. 2006;21(6):459–68.
